# An Assessment of Nutrition Practices and Attitudes in Family Child-Care Homes: Implications for Policy Implementation

**DOI:** 10.5888/pcd12.140587

**Published:** 2015-06-04

**Authors:** Alison Tovar, Patricia Risica, Noereem Mena, Eliza Lawson, Angela Ankoma, Kim M. Gans

**Affiliations:** Author Affiliations: Patricia Risica, Kim M. Gans, Institute for Community Health Promotion, Brown University School of Public Health, Providence, Rhode Island, and Human Development and Family Studies Department and Center for Health, Intervention, and Prevention, University of Connecticut, Storrs, Connecticut; Noereem Mena, Department of Nutrition and Food Sciences, University of Rhode Island, Kingston, Rhode Island; Eliza Lawson, Chronic Disease Prevention and Control Initiative, Rhode Island Department of Health, Providence, Rhode Island; Angela Ankoma, Office of Minority Health, Rhode Island Department of Health, Providence, Rhode Island.

## Abstract

**Introduction:**

Family child-care homes (FCCHs) provide care and nutrition for millions of US children, including 28% in Rhode Island. New proposed regulations for FCCHs in Rhode Island require competencies and knowledge in nutrition. We explored nutrition-related practices and attitudes of FCCH providers in Rhode Island and assessed whether these differed by provider ethnicity or socioeconomic status of the enrolled children.

**Methods:**

Of 536 licensed FCCHs in Rhode Island, 105 randomly selected FCCH providers completed a survey about provider nutrition attitudes and practices, demographics of providers, and characteristics of the FCCH, including participation in the federal Child and Adult Care Food Program (CACFP). No differences between CACFP and non-CACFP participants were found; responses were compared by provider ethnicity using χ^2^ tests and multivariate models.

**Results:**

Nearly 70% of FCCHs reported receiving nutrition training only 0 to 3 times during the past 3 years; however, more than 60% found these trainings to be very helpful. More Hispanic than non-Hispanic providers strongly agreed to sitting with children during meals, encouraging children to finish their plate, and being involved with parents on the topics of healthy eating and weight. These differences persisted in multivariate models.

**Discussion:**

Although some positive practices are in place in Rhode Island FCCHs, there is room for improvement. State licensing requirements provide a foundation for achieving better nutrition environments in FCCHs, but successful implementation is key to translating policies into real changes. FCCH providers need culturally and linguistically appropriate nutrition-related training.

## Introduction

Close to one-third of US children aged 2 to 5 years are overweight or obese. Clear disparities are observed by ethnicity; 17% of Hispanic children in this age group are obese compared with 3.5% of their non-Hispanic white counterparts (1). Contributing to the obesity epidemic are unhealthy eating patterns, including high consumption of energy-dense snack foods and low consumption of fruits and vegetables (2). These patterns are troubling, given that early childhood is a critical period during which dietary intake patterns and eating habits are developed (3). Although parents play a critical role in shaping children’s food preferences and determining their physical and social environments, the child-care setting (nonparental care either at a center or family child-care home [FCCH]) and its providers (those who care for children in child-care settings) are also important in shaping healthy behaviors. Child-care providers can affect children’s healthy eating habits through appropriate feeding practices and attitudes and by influencing the access and availability of healthy foods and beverages in child-care environments (the physical and social conditions at the child-care setting) (4). Therefore, fostering effective strategies to help child-care providers establish healthy eating habits and promote healthy environments among disadvantaged populations is critical.

Creating healthy child-care environments is of great importance, because almost 60% of children in the United States younger than 6 are enrolled in some type of nonparental care each week, and nearly 50% of enrolled children identify as Hispanic (5). Policies and regulations ensure healthy nutrition environments in child-care settings (6), and practical professional training and education of child-care providers is needed to translate policies into healthy practices. Most recent training programs and interventions to improve healthy behaviors focused on child-care centers (7); however, 2 million US children (25%) and 21,000 Rhode Island children (28%) in nonparental care attend an FCCH (8), a child-care setting where children are cared for by child-care providers in their homes rather than in a center. Regulations for licensed FCCHs are different, and in most cases less stringent, than those for free-standing child-care centers (9). Moreover, time spent in FCCH settings during infancy is associated with increased body mass index (BMI) *z*-scores at 3 years of age, and time spent in child-care centers is not (10).

In Rhode Island, recently proposed updated regulations for both child-care centers and FCCHs would require more provider knowledge and competencies, including more nutrition education. For FCCHs, these proposed regulations would include increasing the required hours of provider professional development related to the new competency requirements. These regulations are still under review; however, given that more nutrition training will likely be required, it is important to know how to tailor the content and format of these trainings so that they meet the needs of FCCH providers.

Given the lack of research on FCCHs, understanding provider’s nutrition-related practices and attitudes and exploring variation across contexts is important. With this information, more appropriate trainings relevant to nutrition-related regulatory policies in FCCHs can be developed. The goal of this study was to explore nutrition-related practices and attitudes of FCCH providers in Rhode Island and assess whether these differed by provider ethnicity or socioeconomic status of the enrolled children.

## Methods

Key informant interviews were conducted during summer 2012 with child-care stakeholders from Rhode Island, including FCCH providers and state agency representatives, to inform the development of a statewide survey with Rhode Island FCCH providers. Previous literature on similar evaluation instruments was also reviewed (11–13). On the basis of this formative research, a survey instrument was developed to administer to Rhode Island FCCH providers. The instrument contained 12 home-specific questions including the respondents’ background in education and child-care and whether the center participated in the US Department of Agriculture’s Child and Adult Care Food Program (CACFP), a federal program that provides reimbursement for healthful meals and snacks served to low-income children and adults, and BrightStars, the Rhode Island child-care provider quality rating system (14), both of which include nutrition standards. The survey also included 62 food or nutrition attitude or practice-related questions. Selected food questions were adapted from the California Child-Care Food Assessment’s *Survey of Child Care Providers of 2–5 Year Old Children* (12). For the purpose of this article, we include questions relating to nutrition training, child feeding practices and attitudes, and parental involvement (Table).

**Table:** Provider Responses Related to Nutrition Training, Feeding Practices, and Attitudes About Parent Involvement

Question	All Providers, %				Non-Hispanic Providers, %				Hispanic Providers, %				Hispanic, Crude	Hispanic, Adjusted for CACFP
**Nutrition Training**														
**No. of times**	**0–3**		**4–7**		**0–3**		**4–7**		**0–3**		**4–7**		**β (*P*)**	**β (*P*)**
How often have you received training on nutrition in the past 3 years?	67.6		32.4		76.2		23.8		53.9		46.2		−1.0 (.02)	−1.1 (.02)
**Level of helpfulness**	**N/S**		**Very**		**N/S**		**Very**		**N/S**		**Very**		**β (*P*)**	**β (*P*)**
How helpful did you find the class(es)?	35.2		64.8		54.9		45.1		8.1		91.9		−2.6 (<.001)	−2.4 (<.001)
How helpful do you think it would be to have more training focused on home based child-care programs?	42.4		57.6		63.8		36.2		12.2		87.8		−2.5 (<.001)	−2.5 (<.001)
**Level of agreement**	**SA**	**A**	**D**	**SD**	**SA**	**A**	**D**	**SD**	**SA**	**A**	**D**	**SD**	**β (*P*)**	**β (*P*)**
I regularly attend nutrition education classes.	41.2	30	24.7	4.1	27.6	25.9	39.7	6.9	61.5	35.9	24.7	4.1	−1.4 (.001)	−1.1 (.03)
**Provider Feeding Practices^a^**														
I sit with the children during snacks and meals.	67.3	29.8	1.0	1.9	59.4	35.9	1.6	3.1	80.0	20.0	0	0	−1.0 (.03)	−1.1 (.02)
I rarely eat the same foods and have the same drinks as the children.	9.8	24.5	40.2	25.5	6.4	33.3	39.7	20.6	15.4	10.3	41.0	33.3	−0.46 (.22)	−0.65 (.11)
I am highly motivated to serve healthy foods to the children.	71.8	28.2	—	—	58.7	41.3	—	—	92.5	7.5	—	—	2.2 (.001)	1.9 (.006)
Children are encouraged to finish the food on their plate.	38.8	35.9	18.5	6.8	12.7	49.2	28.6	9.5	80.0	15.0	2.5	2.5	−3.3 (<.001)	−3.1 (<.001)
Children are involved in nutrition-related plans, books, and activities.	2.0	7.9	46.5	43.5	3.2	8.0	53.2	35.5	0	7.7	35.9	56.4	−0.86 (.04)	−0.94 (.04)
Children are involved in cooking and hands-on food activities.	6.8	8.7	56.3	28.2	7.9	9.5	52.4	30.2	5.0	7.5	62.5	25.0	0.39 (.50)	0.42 (.49)
Children like to try new foods.	19.8	53.5	18.8	7.9	15.9	46.0	25.4	12.7	26.3	65.8	7.9	0	−0.64 (.21)	−0.5 (.30)
**Attitudes About Parents/Parent Involvement^a^**														
Parents/families demand that their children be served healthy foods.	22.8	45.5	21.8	9.9	9.7	53.2	25.8	11.3	43.6	33.3	15.4	7.7	0.68 (.14)	0.66 (.17)
From conversations with parents/families and children, it seems that parents know about healthy foods to serve to children.	18.2	58.6	19.2	4.0	12.5	60.9	20.3	6.3	28.6	54.3	17.1	0	0.56 (.29)	0.24 (.66)
From conversations with parents/families and children, it seems that parents reinforce nutrition education at home.	10.3	52.6	28.9	8.3	5.0	56.7	28.3	10.0	18.9	46.0	29.7	5.4	0.14 (.75)	0.09 (.84)
It seems that parents/families are concerned about the quality of fruits and vegetables.	16.0	50.0	24.5	9.6	6.9	51.7	27.6	13.8	30.6	47.2	19.4	2.8	0.9 (.06)	0.86 (.09)
When parents/families bring outside meals for their children, the food brought in is often healthy.	7.2	48.2	24.1	20.5	8.9	48.2	26.8	16.0	3.7	48.2	18.5	29.6	−0.21 (.65)	0.05 (.92)
Parents/families regularly ask what their child has eaten.	27.7	45.5	20.8	5.9	20.6	47.6	23.8	7.9	39.5	42.1	15.8	2.6	0.72 (.15)	0.43 (.42)
A parent has stated that their children will eat certain foods at daycare and not at home.	40.4	38.4	14.1	7.1	42.9	46.0	4.8	6.4	36.1	25.0	30.6	8.3	−1.6 (.002)	−1.5 (.005)
I believe it is important to communicate with parents/families regarding nutrition.	63.5	31.7	4.8	—	47.6	46.0	6.3	—	87.8	9.8	2.4	—	−1.9 (<.001)	−1.5 (.003)
I discuss with parents/families if lunches or snacks sent in are not healthy	38.9	41.1	16.7	3.3	20.4	50.0	24.0	5.6	66.7	27.8	5.6	0	−1.3 (.001)	−1.3 (<.001)
It seems to me that parents/families give up too easily when trying to feed healthy food to their children.	35.7	39.8	19.4	5.1	23.3	46.7	23.3	6.7	55.3	29.0	16.2	2.6	−0.36 (.32)	−0.33 (.38)
I am comfortable passing information on to parents/families about good nutrition practices.	54.9	43.1	2.0	—	45.2	51.6	3.2	—	70.0	30.0	0	—	1.0 (.01)	1.0 (.02)
I am comfortable encouraging parents/families to breastfeed their infants.	67.8	20.0	7.8	4.4	59.6	19.2	13.5	7.7	79.0	21.0	0	0	1.2 (.003)	1.2 (.008)
I am comfortable discussing a child’s weight problem with parents/families.	36.5	37.5	20.8	5.2	22.8	36.8	31.6	8.8	56.4	38.5	5.1	0	0.74 (.05)	0.59 (.12)

Abbreviations: A, agree; CACFP, Child and Adult Care Food Program; D, disagree; N/S, not/somewhat; SA, strongly agree; SD, strongly disagree.

a Response options for some variables were collapsed because of small numbers; more information about collapsing of variables can be obtained from the corresponding author.

We set a goal of obtaining 100 completed surveys from the 536 licensed FCCHs in Rhode Island. Because the feasibility and cost of reaching all 536 licensed FCCHs was prohibitive, we determined that reaching 100 licensed providers was an adequate number from which to draw conclusions. Calls were made to batches of 10 to 15 numbers at a time, which were called until a final disposition was determined before working on a new batch of numbers. This method ensured that all numbers received the same attention and minimized the bias from assessing only early responders. The team stopped calling new batches when the goal of 100 completed surveys was close to being reached, which happened as we completed the twelfth batch. Our sample from the 12 batches included 360 providers; 243 of these were eligible (still in business, spoke English or Spanish). FCCH providers who were reached were offered to take the survey by telephone, online, or receive it as a paper document to be returned in the mail. The study was reviewed and approved by the institutional review board at Brown University, Providence, Rhode Island.

Chi-square statistics were calculated to determine differences in reported practices and attitudes by CACFP and provider ethnicity status in separate analyses. Few of the items differed by CACFP status, so the multivariate analysis focused on ethnic differences. Multivariate models were constructed by using SAS version 9.3 (SAS Institute, Inc) as generalized linear models using a logit link function and indicating that the response options were multinomial. Because of small numbers in some cells, response options were collapsed for some variables (Table). Final models included CACFP status as an adjustment for both program participation and as a proxy indicator of socioeconomic status of the provider and children being served.

## Results

A total of 105 FCCH providers, representing 43% of the eligible FCCHs called, completed a survey in either Spanish (35%) or English (65%). Ineligible homes (33% of the overall sample called) were those in which the telephone number was not working or the home was no longer a child-care home provider. Of the responding providers, 39% identified as Hispanic, 43% as non-Hispanic white, and 3% as non-Hispanic black. Providers estimated the families of children in their care as 43% Hispanic, 46% non-Hispanic white, 7% non-Hispanic black, and 2% Asian. Half (50%) of responding providers indicated that they participated in CACFP, and 34% reported participating in BrightStars.

Nearly 70% of providers reported receiving nutrition training only 0 to 3 times during the past 3 years; however, more than 60% of them found these trainings to be very helpful. They also reported that it would be very helpful to have more nutrition training specific for FCCHs (Table). Most providers strongly agreed that they sit with children during snacks and meals (67.3%) and were highly motivated to serve healthy foods to children (71.8%). Approximately three-quarters, however, agreed or strongly agreed that they encourage children to finish all the food on their plate (74.7%). Most providers (90%) disagreed or strongly disagreed that the children in their care were involved in nutrition-related plans and activities.

Several significant differences were observed by provider ethnicity, even after adjustment for CACFP status. Hispanic providers reported receiving more nutrition training during the past 3 years than did non-Hispanic providers (46.2% vs 23.8%) and were more likely to find the training very helpful (91.9% vs 45.1%) (Table). More Hispanic providers reported strongly agreeing to sitting with children during snacks and meals than did non-Hispanic providers (80.0% vs 59.4%, *P* = .02), but more Hispanic providers also reported strongly agreeing to encourage children to finish food on their plate than did non-Hispanic providers (80.0% vs 12.7%, *P* < .001). Hispanic providers also reported less child involvement in nutrition-related plans and activities than did non-Hispanic providers (56.4% vs 35.5%, *P* = .04) (Table).

More non-Hispanic than Hispanic providers strongly agreed or agreed with the statement that “parents say their children will eat certain foods at daycare and not at home” (88.9% vs 61.1%). More Hispanic than non-Hispanic providers strongly agreed with the following statements: “I believe it is important to communicate with parents/families regarding nutrition” (87.8% vs 46.9%) and “I discuss with parents/families if lunches or snacks sent in are not healthy” (66.7% vs 20.4%). Hispanic FCCH providers also felt more comfortable than non-Hispanic providers in passing information on to parents and families about good nutrition practices (70.0% vs 45.2%) and encouraging breastfeeding (79.0% vs 59.6%). More Hispanic than non-Hispanic providers strongly agreed that they were comfortable discussing a child’s weight problem with parents or families (56.4% vs 22.8%) (Table).

## Discussion

The goals of this study were to explore provider practices and attitudes related to nutrition in a sample of Rhode Island FCCH providers and to assess if practices differed by ethnicity of the provider. Overall, we found that positive practices were displayed related to feeding, such as sitting with the children during meals and snacks and eating the same foods as the children, which provide an opportunity to model appropriate behaviors and positive feeding practices with children (15). However, we also found providers commonly encouraged children to finish all the food on their plates, which may interfere with a child’s internal cues for satiety and hunger, possibly contributing to the development of obesity (16). Controlling feeding practices are associated with the development of unhealthy eating behaviors and childhood obesity (16). Training for FCCH providers should address responsive child-feeding practices (17), including allowing children to control the amount of food they eat without pressure or control, modeling healthy eating, and serving meals family-style.

Although we hypothesized that nutrition practices may differ by CACFP program participation as a proxy for socioeconomic status, we found that this was not the case. However, provider ethnicity was a predictor of certain nutrition practices, suggesting a possible cultural influence among these providers. More Hispanic than non-Hispanic providers reported sitting with children while they ate, but Hispanic providers also reported being more likely to encourage children to finish all the food on their plate and less likely to involve children in nutrition education. Hispanic providers also reported more communication with parents about children’s diet and weight. Results from several studies have shown that feeding practices differ by ethnicity (18). For example, some studies indicate that Hispanic parents are less likely to set limits around meal times compared with other racial/ethnic groups, although results are mixed (19). Although most of these studies were conducted with parents instead of child-care providers, one study found that Hispanic providers were more involved than non-Hispanic providers with what the children were doing during mealtimes and exhibited more demanding practices such as making children eat all the food on their plate (20). Another study also supports our findings in that Hispanic providers (both home- and center-based) were more likely to encourage children to finish meals (21). Given that many FCCHs serve ethnic minority children and are run by ethnically diverse providers (in Rhode Island, more than 40% of FCCH providers are Spanish-speaking), these findings highlight possible cultural differences and underscore the need for policies and trainings to be culturally relevant. Our findings also indicate the importance of training non-Hispanic providers about responsive feeding, including sitting with children during meals. Further research is needed to better understand providers’ feeding practices and explore how continuing education and training can be delivered so that providers can learn how to improve feeding practices in culturally appropriate ways. In child-care centers, training providers in nutrition practices has improved provider knowledge, center policies, and children’s diet quality and weight status (6,22), but more research is needed on such interventions with FCCHs.

Our data also showed that more Hispanic providers than non-Hispanic providers felt comfortable communicating with parents about healthy foods and a healthy weight. Although evidence suggests that parents who use an FCCH appreciate a more intimate relationship with providers (20), our results suggest that in this population there are ethnic variations and that Hispanic providers may feel a closer relationship with parents. Hispanics tend to be more collectivistic, family-oriented, and focused on maintaining smooth and positive social interactions than non-Hispanic whites (23). These cultural norms may allow for a more open discussion between parents and FCCH providers about children’s eating habits and weight status. Having this open line of communication is important for Hispanic parents, because they frequently do not recognize their children as overweight and may have a more favorable view of childhood obesity, which may prevent them from seeing their child’s weight as problematic (24). Both Hispanic providers and parents may be less concerned about preventing childhood obesity and more concerned about their child eating enough (25). Future research should examine these issues and explore ways to facilitate better communication about nutrition between providers and parents, especially among non-Hispanic providers. A previous study found that providers reported the need for better communication and cooperation with parents (26). Prior studies have also emphasized the need to influence both the home and the child-care environment to successfully engage in obesity prevention, because children spend time in both these environments (27,28). One study has shown the promise of including parents and home-based activities as part of child-care–based interventions in reducing BMI *z*-scores in young children (29). Therefore, future work should use an ecological approach when exploring the interactions between home and child-care environments and how positive obesity prevention practices and environments can be consistent across both settings.

Regardless of provider ethnicity, our findings suggest the need for effective policies and supportive trainings and resources to improve the nutrition environments of FCCHs. Many expert groups (30–32) have emphasized the establishment of quality prevention policies as a foundation for improving the food, physical activity, breastfeeding, and screen-time environments in child-care settings (33). However, creating policies alone is not enough (30). Policies are more likely to succeed with trainings and education for child-care providers (34), and few studies have documented FCCH provider training. We found that most FCCH providers do not frequently participate in nutrition training, which is similar to the findings of Trost et al who found that less than 50% of staff in FCCHs received annual training on nutrition (35). Although provider participation in Rhode Island FCCH trainings is low, interest is high; more than 50% of FCCH providers in this study indicated that they were interested in further training, especially training that is focused on their specific needs. Such training should include practical examples of how to implement changes in food and physical activity environments, as well as motivational examples of successful changes in FCCHs with compelling role models. These trainings should also educate providers on responsive feeding practices and how they could involve children in food preparation and education.

Our study has limitations. First, we used self-reported data, which were not validated and may have been influenced by socially desirable responses. However, this bias would have affected the entire sample and would not explain the ethnic differences we observed. A few studies have addressed the issues of self-report and social desirability bias by direct observation of child-care practices; this approach is more objective but time-consuming and cost-intensive. Second, providers who did not respond to the survey despite many attempts may have represented a more time- or resource-constrained group, and those we did reach may have been more health-conscious, which may have introduced selection bias. However, the high proportion of FCCHs reached (43% of those randomly chosen and eligible) indicates that this bias is likely small. Finally, we did not have data on child race/ethnicity and income; rather, participation in CACFP was used as a proxy indicator of socioeconomic status of the provider and children being served.

In conclusion, we found that, although positive practices exist in the FCCHs surveyed, there is room for improvement. It is important for FCCHs to follow practices that are consistent with the 2011 national recommendations for child-care policies and practices to reduce childhood obesity (36). Rhode Island recently proposed strengthening its regulations for child-care centers and FCCHs (including health and nutrition) (14), although these regulations are still under review. However, even if such policies and regulations are strengthened, FCCH providers are unlikely to follow them without adequate training and resources. Our findings will inform the development of new trainings that incorporate information of the recently proposed regulations. These trainings can also be enhanced to include information on responsive feeding and parent communication and ensure that they are culturally and linguistically appropriate for ethnically diverse FCCH providers. Our results will be communicated to state and local agencies and organizations such as the Department of Children, Youth and Families; Rhode Island Department of Education; the Center for Early Learning Professionals; and Ready to Learn Providence to enable such stakeholders to work together to translate policies, regulations, or quality rating systems into practical and effective trainings that are appropriate for different racial and ethnic groups.
